# Tetra-μ-acetato-bis­[(pyridine *N*-oxide)copper(II)](*Cu*—*Cu*)

**DOI:** 10.1107/S1600536809024222

**Published:** 2009-07-01

**Authors:** Yue Cui, Qian Gao, Chao-Yan Zhang, Ya-Bo Xie

**Affiliations:** a College of Environmental and Energy Engineering, Beijing University of Technology, Beijing 100124, People’s Republic of China

## Abstract

The mol­ecule of the title binuclear copper(II) complex, [Cu_2_(CH_3_COO)_4_(C_5_H_5_NO)_2_], occupies a special position on a crystallographic inversion centre; the coordination environment of the Cu^II^ atom is slightly distorted square-pyramidal and is made up of four O atoms belonging to four acetate groups in the basal plane with the O atom of pyridine *N*-oxide ligand in the apical position. The Cu—Cu distance is 2.6376 (6) Å.

## Related literature

For the biological activity of binuclear copper(II) compounds, see: Li *et al.* (2007 ▶). For a related structure, see: Zhang (2009 ▶).
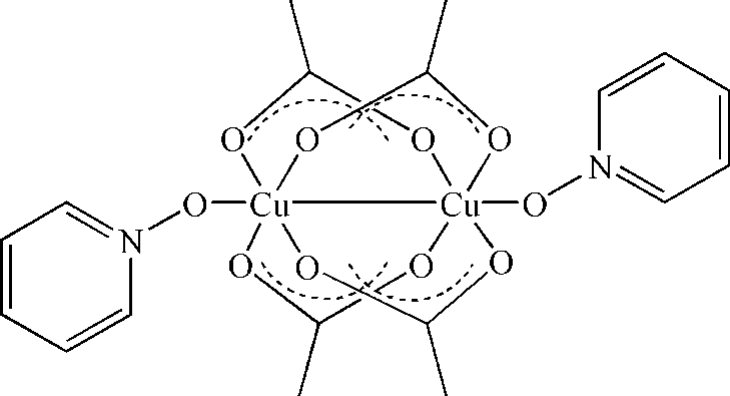


## Experimental

### 

#### Crystal data

[Cu_2_(C_2_H_3_O_2_)_4_(C_5_H_5_NO)_2_]
*M* *_r_* = 553.46
Monoclinic, 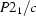

*a* = 9.6737 (11) Å
*b* = 13.5886 (16) Å
*c* = 8.5236 (10) Å
β = 99.970 (2)°
*V* = 1103.5 (2) Å^3^
*Z* = 2
Mo *K*α radiation
μ = 1.98 mm^−1^
*T* = 296 K
0.2 × 0.2 × 0.2 mm

#### Data collection

Bruker SMART CCD area-detector diffractometer
Absorption correction: multi-scan (*SADABS*; Bruker, 1998 ▶) *T*_min_ = 0.673, *T*_max_ = 0.680
5445 measured reflections
1936 independent reflections
1713 reflections with *I* > 2σ(*I*)
*R*_int_ = 0.015

#### Refinement

*R*[*F* ^2^ > 2σ(*F* ^2^)] = 0.025
*wR*(*F* ^2^) = 0.070
*S* = 1.07
1936 reflections
145 parameters
H-atom parameters constrained
Δρ_max_ = 0.43 e Å^−3^
Δρ_min_ = −0.33 e Å^−3^

### 

Data collection: *SMART* (Bruker, 1998 ▶); cell refinement: *SAINT* (Bruker, 1998 ▶); data reduction: *SAINT*; program(s) used to solve structure: *SHELXS97* (Sheldrick, 2008 ▶); program(s) used to refine structure: *SHELXL97* (Sheldrick, 2008 ▶); molecular graphics: *SHELXTL* (Sheldrick, 2008 ▶); software used to prepare material for publication: *SHELXTL*.

## Supplementary Material

Crystal structure: contains datablocks global, I. DOI: 10.1107/S1600536809024222/ya2100sup1.cif

Structure factors: contains datablocks I. DOI: 10.1107/S1600536809024222/ya2100Isup2.hkl

Additional supplementary materials:  crystallographic information; 3D view; checkCIF report

## Figures and Tables

**Fig. 1.**
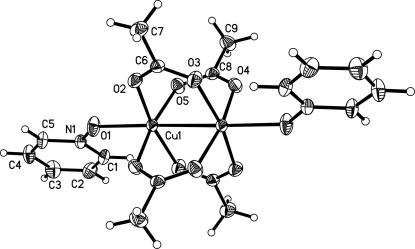
Molecular structure of the title compound with displacement ellipsoids drawn at the 30% probability level; hydrogen atoms are shown as small circles of arbitrary radius. The unlabelled atoms are derived by the symmetry transformation -x + 1, -y, -z.

## supplementary crystallographic information

### Comment

The crystal structures of binuclear copper(II) complexes have been extensively studied because of their possible anticarcinogen properties (Li *et al.*, 2007), and numerous papers dealing with binuclear copper complexes have been published (Zhang, 2009). Herein, we report the synthesis and crystal structure of a new binuclear copper complex.

The molecule of the title binuclear copper(II) complex, [Cu_2_(C_2_H_3_O_2_)_4_(C_5_H_5_ON)_2_], occupies a special position in the crystallographic inversion centre; coordination environment of the Cu^II^ atom represents a slightly distorted tetragonal pyramid and is made up of four oxygen atoms belonging to four acetato-group in the basal plane as well as the oxygen atom of pyridine N-oxide ligand in the apical position. The Cu—O bond distance between Cu^II^ atom and acetato O atoms vary from 1.9605 (18) Å to 1.9710 (18) Å, while the Cu—O bond distance involving Cu^II^ atom and the O atom of the pyridine N-oxide ligand is 2.1507 (18) Å. The Cu1—Cu1^i^ distance is 2.6376 (6) Å [symmetry code (i): 1 - *x*, -*y*, -*z*].

### Experimental

A solution containing a 1:2:5 molar ratio of picolinic acid N-oxide (0.0139 g, 0.1 mmol), CuCO_3_ (0.0247 g, 0.2 mmol) and acetic acid (1 ml, 0.5 mmol/ml) in a mixture of ethanol(5 ml) and water (10 ml) was sealed in a 25 ml teflon reactor and kept at 453 K for 3 days, then slowly cooled to 373 k and kept at this temperature for 24 h more. After cooling to room temperature, the mixture was filtered and the filtrate was allowed to stand at room temperature. Block crystals suitable for the X-ray investigation were collected.

### Refinement

All H atoms were placed geometrically (C-H = 0.93-0.96 Å) and included into refinement in the riding motion approximation with U_iso_(H) = 1.2U_eq_(C) [1.5U_eq_(C) for methyl H atoms].

### Crystal data

**Table:**

[Cu_2_(C_2_H_3_O_2_)_4_(C_5_H_5_NO)_2_]	*F*(000) = 564
*M**_r_* = 553.46	*D*_x_ = 1.666 Mg m^−^^3^
Monoclinic, *P*2_1_/*c*	Mo *K*α radiation, λ = 0.71073 Å
Hall symbol: -P 2ybc	Cell parameters from 3911 reflections
*a* = 9.6737 (11) Å	θ = 2.6–27.9°
*b* = 13.5886 (16) Å	µ = 1.98 mm^−^^1^
*c* = 8.5236 (10) Å	*T* = 296 K
β = 99.970 (2)°	Block, blue
*V* = 1103.5 (2) Å^3^	0.2 × 0.2 × 0.2 mm
*Z* = 2	

### Data collection

**Table:**

Bruker SMART CCD area-detector diffractometer	1936 independent reflections
Radiation source: fine-focus sealed tube	1713 reflections with *I* > 2σ(*I*)
graphite	*R*_int_ = 0.015
φ and ω scans	θ_max_ = 25.0°, θ_min_ = 2.1°
Absorption correction: multi-scan (*SADABS*; Bruker, 1998)	*h* = −10→11
*T*_min_ = 0.673, *T*_max_ = 0.680	*k* = −16→9
5445 measured reflections	*l* = −10→9

### Refinement

**Table:**

Refinement on *F*^2^	Primary atom site location: structure-invariant direct methods
Least-squares matrix: full	Secondary atom site location: difference Fourier map
*R*[*F*^2^ > 2σ(*F*^2^)] = 0.025	Hydrogen site location: inferred from neighbouring sites
*wR*(*F*^2^) = 0.070	H-atom parameters constrained
*S* = 1.07	*w* = 1/[σ^2^(*F*_o_^2^) + (0.0352*P*)^2^ + 0.7718*P*] where *P* = (*F*_o_^2^ + 2*F*_c_^2^)/3
1936 reflections	(Δ/σ)_max_ = 0.001
145 parameters	Δρ_max_ = 0.43 e Å^−^^3^
0 restraints	Δρ_min_ = −0.33 e Å^−^^3^

### Special details

**Table:**

Geometry. All e.s.d.'s (except the e.s.d. in the dihedral angle between two l.s. planes) are estimated using the full covariance matrix. The cell e.s.d.'s are taken into account individually in the estimation of e.s.d.'s in distances, angles and torsion angles; correlations between e.s.d.'s in cell parameters are only used when they are defined by crystal symmetry. An approximate (isotropic) treatment of cell e.s.d.'s is used for estimating e.s.d.'s involving l.s. planes.
Refinement. Refinement of *F*^2^ against ALL reflections. The weighted *R*-factor *wR* and goodness of fit *S* are based on *F*^2^, conventional *R*-factors *R* are based on *F*, with *F* set to zero for negative *F*^2^. The threshold expression of *F*^2^ > σ(*F*^2^) is used only for calculating *R*-factors(gt) *etc*. and is not relevant to the choice of reflections for refinement. *R*-factors based on *F*^2^ are statistically about twice as large as those based on *F*, and *R*- factors based on ALL data will be even larger.

### Fractional atomic coordinates and isotropic or equivalent isotropic displacement parameters (Å^2^)

**Table:**

	*x*	*y*	*z*	*U*_iso_*/*U*_eq_	
O3	0.55087 (19)	−0.15687 (13)	−0.0542 (2)	0.0457 (5)	
O4	0.69513 (18)	−0.02840 (14)	0.1692 (2)	0.0411 (4)	
Cu1	0.38549 (3)	0.01927 (2)	0.05882 (3)	0.02846 (12)	
O5	0.50246 (19)	0.00354 (14)	0.2694 (2)	0.0424 (4)	
O2	0.35940 (18)	−0.12383 (13)	0.0480 (2)	0.0408 (4)	
N1	0.11758 (19)	0.11134 (15)	0.1808 (2)	0.0330 (4)	
C8	0.6302 (3)	−0.01859 (17)	0.2828 (3)	0.0335 (5)	
O1	0.18110 (19)	0.03795 (15)	0.1215 (3)	0.0520 (5)	
C6	0.4409 (3)	−0.18155 (18)	−0.0051 (3)	0.0349 (5)	
C1	0.1856 (3)	0.1945 (2)	0.2326 (3)	0.0409 (6)	
H1A	0.2801	0.2014	0.2261	0.049*	
C5	−0.0186 (2)	0.1004 (2)	0.1894 (3)	0.0406 (6)	
H5A	−0.0653	0.0428	0.1529	0.049*	
C9	0.7115 (3)	−0.0358 (2)	0.4475 (3)	0.0506 (7)	
H9A	0.8070	−0.0518	0.4408	0.076*	
H9B	0.6700	−0.0892	0.4966	0.076*	
H9C	0.7096	0.0228	0.5102	0.076*	
C2	0.1170 (3)	0.2692 (2)	0.2948 (3)	0.0499 (7)	
H2A	0.1650	0.3265	0.3302	0.060*	
C4	−0.0892 (3)	0.1741 (2)	0.2519 (4)	0.0509 (7)	
H4A	−0.1835	0.1658	0.2581	0.061*	
C3	−0.0225 (3)	0.2599 (2)	0.3054 (4)	0.0545 (8)	
H3A	−0.0702	0.3102	0.3474	0.065*	
C7	0.4047 (3)	−0.2887 (2)	−0.0104 (4)	0.0530 (7)	
H7A	0.3190	−0.2983	0.0300	0.080*	
H7B	0.4789	−0.3249	0.0539	0.080*	
H7C	0.3930	−0.3117	−0.1184	0.080*	

### Atomic displacement parameters (Å^2^)

**Table:**

	*U*^11^	*U*^22^	*U*^33^	*U*^12^	*U*^13^	*U*^23^
O3	0.0419 (10)	0.0288 (9)	0.0715 (13)	−0.0013 (8)	0.0238 (9)	−0.0027 (9)
O4	0.0337 (9)	0.0532 (12)	0.0363 (9)	0.0075 (8)	0.0062 (7)	0.0057 (8)
Cu1	0.02487 (17)	0.02651 (18)	0.03512 (19)	0.00156 (11)	0.00833 (12)	0.00071 (11)
O5	0.0362 (10)	0.0558 (12)	0.0356 (9)	0.0061 (8)	0.0071 (7)	−0.0015 (8)
O2	0.0409 (10)	0.0286 (9)	0.0557 (11)	−0.0019 (7)	0.0158 (8)	0.0008 (8)
N1	0.0279 (10)	0.0374 (11)	0.0350 (10)	−0.0006 (9)	0.0088 (8)	−0.0034 (9)
C8	0.0374 (13)	0.0264 (12)	0.0364 (13)	−0.0007 (10)	0.0055 (10)	0.0015 (10)
O1	0.0339 (10)	0.0484 (12)	0.0788 (14)	−0.0019 (8)	0.0237 (9)	−0.0203 (10)
C6	0.0356 (13)	0.0277 (12)	0.0402 (13)	−0.0017 (10)	0.0034 (10)	0.0044 (10)
C1	0.0319 (13)	0.0444 (15)	0.0470 (15)	−0.0119 (11)	0.0089 (11)	−0.0035 (12)
C5	0.0275 (12)	0.0444 (15)	0.0512 (15)	−0.0077 (11)	0.0106 (11)	−0.0127 (12)
C9	0.0508 (16)	0.0627 (19)	0.0368 (15)	0.0086 (14)	0.0031 (12)	0.0055 (13)
C2	0.0540 (17)	0.0408 (15)	0.0554 (18)	−0.0122 (13)	0.0108 (13)	−0.0108 (13)
C4	0.0308 (14)	0.0589 (18)	0.0654 (18)	−0.0008 (12)	0.0146 (12)	−0.0178 (15)
C3	0.0526 (17)	0.0506 (18)	0.0626 (19)	0.0068 (14)	0.0162 (14)	−0.0180 (15)
C7	0.0568 (18)	0.0282 (14)	0.077 (2)	−0.0029 (12)	0.0187 (15)	0.0036 (14)

### Geometric parameters (Å, °)

**Table:**

O3—C6	1.254 (3)	C1—C2	1.369 (4)
O4—C8	1.250 (3)	C1—H1A	0.9300
Cu1—O5	1.9605 (18)	C5—C4	1.370 (4)
Cu1—O2	1.9610 (18)	C5—H5A	0.9300
Cu1—O4^i^	1.9685 (17)	C9—H9A	0.9600
Cu1—O3^i^	1.9710 (18)	C9—H9B	0.9600
Cu1—O1	2.1507 (18)	C9—H9C	0.9600
Cu1—Cu1^i^	2.6376 (6)	C2—C3	1.373 (4)
O5—C8	1.257 (3)	C2—H2A	0.9300
O2—C6	1.251 (3)	C4—C3	1.372 (4)
N1—O1	1.317 (3)	C4—H4A	0.9300
N1—C5	1.340 (3)	C3—H3A	0.9300
N1—C1	1.344 (3)	C7—H7A	0.9600
C8—C9	1.504 (3)	C7—H7B	0.9600
C6—C7	1.496 (3)	C7—H7C	0.9600
			
C6—O3—Cu1^i^	123.11 (16)	O3—C6—C7	117.3 (2)
C8—O4—Cu1^i^	126.30 (16)	N1—C1—C2	120.5 (2)
O5—Cu1—O2	89.09 (8)	N1—C1—H1A	119.7
O5—Cu1—O4^i^	167.89 (7)	C2—C1—H1A	119.7
O2—Cu1—O4^i^	89.45 (8)	N1—C5—C4	120.1 (2)
O5—Cu1—O3^i^	89.38 (8)	N1—C5—H5A	120.0
O2—Cu1—O3^i^	167.91 (7)	C4—C5—H5A	120.0
O4^i^—Cu1—O3^i^	89.55 (8)	C8—C9—H9A	109.5
O5—Cu1—O1	101.25 (8)	C8—C9—H9B	109.5
O2—Cu1—O1	90.70 (7)	H9A—C9—H9B	109.5
O4^i^—Cu1—O1	90.79 (8)	C8—C9—H9C	109.5
O3^i^—Cu1—O1	101.35 (7)	H9A—C9—H9C	109.5
O5—Cu1—Cu1^i^	86.37 (6)	H9B—C9—H9C	109.5
O2—Cu1—Cu1^i^	83.97 (5)	C1—C2—C3	120.3 (3)
O4^i^—Cu1—Cu1^i^	81.52 (5)	C1—C2—H2A	119.8
O3^i^—Cu1—Cu1^i^	83.97 (5)	C3—C2—H2A	119.8
O1—Cu1—Cu1^i^	170.66 (6)	C5—C4—C3	120.9 (3)
C8—O5—Cu1	120.79 (16)	C5—C4—H4A	119.6
C6—O2—Cu1	123.67 (16)	C3—C4—H4A	119.6
O1—N1—C5	117.7 (2)	C4—C3—C2	117.8 (3)
O1—N1—C1	121.9 (2)	C4—C3—H3A	121.1
C5—N1—C1	120.3 (2)	C2—C3—H3A	121.1
O4—C8—O5	124.9 (2)	C6—C7—H7A	109.5
O4—C8—C9	117.0 (2)	C6—C7—H7B	109.5
O5—C8—C9	118.0 (2)	H7A—C7—H7B	109.5
N1—O1—Cu1	134.01 (15)	C6—C7—H7C	109.5
O2—C6—O3	125.2 (2)	H7A—C7—H7C	109.5
O2—C6—C7	117.5 (2)	H7B—C7—H7C	109.5
			
O2—Cu1—O5—C8	82.77 (19)	O5—Cu1—O1—N1	76.0 (2)
O4^i^—Cu1—O5—C8	−0.3 (5)	O2—Cu1—O1—N1	165.2 (2)
O3^i^—Cu1—O5—C8	−85.24 (19)	O4^i^—Cu1—O1—N1	−105.3 (2)
O1—Cu1—O5—C8	173.30 (19)	O3^i^—Cu1—O1—N1	−15.6 (3)
Cu1^i^—Cu1—O5—C8	−1.25 (18)	Cu1—O2—C6—O3	1.9 (4)
O5—Cu1—O2—C6	−88.0 (2)	Cu1—O2—C6—C7	−178.14 (19)
O4^i^—Cu1—O2—C6	79.9 (2)	Cu1^i^—O3—C6—O2	−0.8 (4)
O3^i^—Cu1—O2—C6	−5.3 (5)	Cu1^i^—O3—C6—C7	179.25 (19)
O1—Cu1—O2—C6	170.7 (2)	O1—N1—C1—C2	−179.5 (2)
Cu1^i^—Cu1—O2—C6	−1.59 (19)	C5—N1—C1—C2	0.0 (4)
Cu1^i^—O4—C8—O5	−3.5 (4)	O1—N1—C5—C4	179.2 (3)
Cu1^i^—O4—C8—C9	175.76 (18)	C1—N1—C5—C4	−0.3 (4)
Cu1—O5—C8—O4	3.1 (3)	N1—C1—C2—C3	0.1 (4)
Cu1—O5—C8—C9	−176.22 (18)	N1—C5—C4—C3	0.5 (5)
C5—N1—O1—Cu1	172.94 (19)	C5—C4—C3—C2	−0.3 (5)
C1—N1—O1—Cu1	−7.5 (4)	C1—C2—C3—C4	0.0 (5)

Symmetry codes: (i) −*x*+1, −*y*, −*z*.
